# SARS-CoV-2 Rebound With and Without Use of COVID-19 Oral Antivirals

**DOI:** 10.15585/mmwr.mm7251a1

**Published:** 2023-12-22

**Authors:** Dallas J. Smith, Anastasia Lambrou, Pragna Patel

**Affiliations:** ^1^Epidemic Intelligence Service, CDC; ^2^CDC COVID-19 Response Team; ^3^Coronavirus and Other Respiratory Viruses Division, National Center for Immunization and Respiratory Diseases, CDC.

**Summary**

**What is already known about this topic?**

Early recommended antiviral treatment prevents hospitalizations and deaths among patients with mild-to-moderate COVID-19 who are at risk for severe disease.

**What is added by this report?**

CDC examined SARS-CoV-2 rebound studies among patients who did and did not receive antiviral treatment. No consistent association between treatment and rebound was identified. The prevalence of rebound varied, depending upon host factors and the definition of rebound. Rebound symptoms were mild. No hospitalizations or deaths occurred from viral rebound.

**What are the implications for public health practice?**

This review suggests that per National Institutes of Health COVID-19 Treatment Guidelines, rebound should not deter providers from prescribing lifesaving antiviral treatments when indicated to prevent morbidity and mortality from COVID-19.

## Abstract

Early treatment with a first-line therapy (nirmatrelvir/ritonavir [Paxlovid] or remdesivir) or second-line therapy (molnupiravir) prevents hospitalization and death among patients with mild-to-moderate COVID-19 who are at risk for severe disease and is recommended by the National Institutes of Health COVID-19 Treatment Guidelines. On May 25, 2023, the Food and Drug Administration approved nirmatrelvir/ritonavir for treatment of adults at high risk for severe disease. Although antiviral therapies are widely available, they are underutilized, possibly because of reports of SARS-CoV-2 rebound after treatment. To enhance current understanding of rebound, CDC reviewed SARS-CoV-2 rebound studies published during February 1, 2020– November 29, 2023. Overall, seven of 23 studies that met inclusion criteria, one randomized trial and six observational studies, compared rebound for persons who received antiviral treatment with that for persons who did not receive antiviral treatment. In four studies, including the randomized trial, no statistically significant difference in rebound rates was identified among persons receiving treatment and those not receiving treatment. Depending on the definition used, the prevalence of rebound varied. No hospitalizations or deaths were reported among outpatients who experienced rebound, because COVID-19 signs and symptoms were mild. Persons receiving antiviral treatment might be at higher risk for rebound compared with persons not receiving treatment because of host factors or treatment-induced viral suppression early in the course of illness. The potential for rebound should not deter clinicians from prescribing lifesaving antiviral treatments when indicated to prevent morbidity and mortality from COVID-19.

## Introduction

COVID-19 has caused approximately 6.5 million hospitalizations and 1.1 million deaths in the United States.* Although hospitalizations and deaths are currently much lower than they were during the peak of the pandemic, COVID-19 continues to cause substantial morbidity and mortality. As of December 9, 2023, approximately 23,000 hospitalizations per week were reported among patients with COVID-19, with highest rates among persons aged ≥65 years. Currently, health care providers are positioned to mitigate COVID-19 morbidity and mortality with safe and effective vaccines^†^ and early diagnosis and treatment (*1*).

### Antiviral Therapeutics

Early treatment with first-line therapy (nirmatrelvir/ritonavir [Paxlovid] or remdesivir) or second-line therapy (molnupiravir) reduces the prevalence of hospitalization and death among patients with mild-to-moderate COVID-19 who are at risk for severe disease (*2*–*4*), and is recommended by the National Institutes of Health (NIH) COVID-19 Treatment Guidelines (*1*). The two oral antivirals, nirmatrelvir/ritonavir and molnupiravir, are widely available but underutilized (*5*). The limited use of these antivirals might be partially attributable to reports of rebound after treatment, especially with nirmatrelvir/ritonavir.^§^ However, rebound was reported before the advent of COVID-19 antivirals and was related to immunity and individual level factors (*6*,*7*).

### SARS-CoV-2 Rebound

SARS-CoV-2 rebound is typically described as recurrence of signs or symptoms or a new positive viral test result after initial recovery from COVID-19. In May 2022, CDC issued a health advisory alert that described case reports of SARS CoV-2 rebound among patients who completed the recommended 5-day course of nirmatrelvir/ritonavir and noted that rebound was also described among persons who were not treated.^¶^ On May 25, 2023, the Food and Drug Administration (FDA) approved nirmatrelvir/ritonavir, which was authorized for emergency use in December 2021, for treatment of mild to moderate COVID-19 among adults aged ≥18 years who are at high risk for severe disease.** In their review of data from Evaluation of Protease Inhibition for COVID-19 in High Risk Patients (EPIC-HR), a phase 2/3 randomized controlled trial that examined the efficacy of nirmatrelvir/ritonavir, FDA concluded that there was no consistent association between treatment and rebound (*8*). To enhance current understanding of rebound, CDC reviewed recent literature comparing rebound among COVID-19 patients who did and did not receive antiviral treatment.

## Review Methodology

**FIGURE 1:**
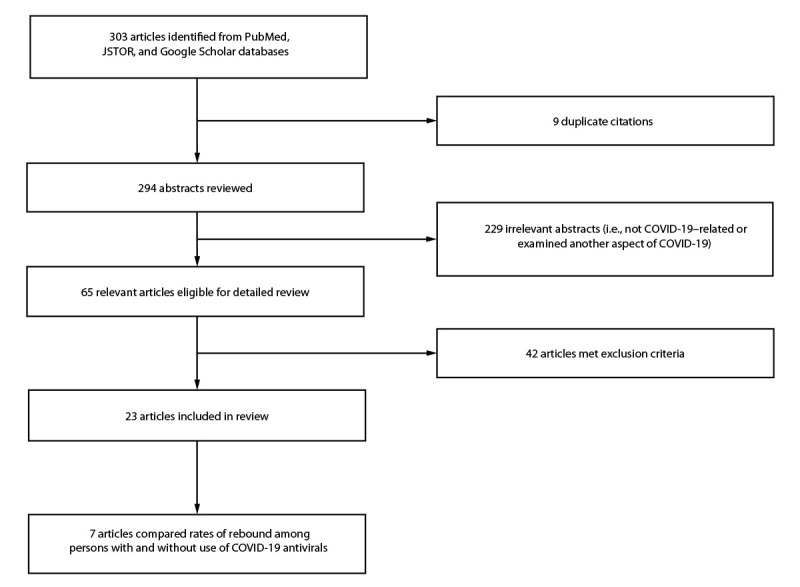
Review of SARS-CoV-2 rebound studies based on specific selection criteria*^,†^ — February 1, 2020–November 29, 2023 * Keywords used in search were, “Paxlovid rebound,” “SARS-CoV-2 viral rebound,” “SARS-CoV-2 rebound,” “nirmatrelvir/ritonavir rebound,” “molnupiravir rebound,” “SARS-CoV-2 infection rebound,” “SARS-CoV-2 viral load rebound,” “rebound phenomenon,” “SARS-CoV-2 viral kinetics,” “SARS-CoV-2 virologic rebound,” and “SARS-CoV-2 clinical rebound.” ^†^ Studies were excluded if they were not related to COVID-19, related to nonrebound aspects of COVID-19, were preprints, editorials, case reports, studies of ancillary medications, or other publications not describing original data or analyses of rebound data.

CDC reviewed SARS-CoV-2 rebound studies published during February 1, 2020–November 29, 2023. The Preferred Reporting Items for Systematic Reviews and Meta-Analyses were used (*9*). PubMed, JSTOR, and Google Scholar were searched using keywords “Paxlovid rebound,” “SARS-CoV-2 viral rebound,” “SARS-CoV-2 rebound,” “nirmatrelvir/ritonavir rebound,” “molnupiravir rebound,” “SARS-CoV-2 infection rebound,” “SARS-CoV-2 viral load rebound,” “rebound phenomenon,” “SARS-CoV-2 viral kinetics,” “SARS-CoV-2 virologic rebound,” and “SARS-CoV-2 clinical rebound.” Searches returned 303 publications that were reviewed^††^ (Figure 1); 23 studies met inclusion criteria (Table 1) (Supplementary Table, https://stacks.cdc.gov/view/cdc/137156). Seven studies compared rates of rebound among patients who did and did not receive COVID-19 antiviral treatment (Table 2) (*10*–*16*). Findings from two studies examining infectivity, resistance, and immune response were summarized (*11*,*17*). Individual case data from three studies that used the same definition of viral rebound were examined to estimate days to onset of viral rebound and rebound duration (*18*–*20*). Median days to rebound and resolution of acute and rebound illness were calculated. Pearson’s chi-square or Fisher’s exact test were used to compare proportions for studies that did not report the test statistic. This activity was reviewed by CDC, deemed not research, and was conducted consistent with applicable federal law and CDC policy.^§§^

**TABLE 1:** SARS-CoV-2 rebound literature review* inclusion and exclusion criteria — February 1, 2020–November 29, 2023

Characteristic	Inclusion criteria	Exclusion criteria
**Publication type**	Peer-reviewed	Preprints Conference abstracts Editorials
**Characteristic**	Published in English Explicitly stated a focus on “rebound” SARS-CoV-2 infection	Published in any other language Not focused on “rebound” All other pathogens such as influenza
**Study type**	Randomized control trials Prospective or retrospective cohort studies Case control studies Case series of two or more patients	Case reports of single patients
**Population**	Adults with SARS-CoV-2 infection	Animal studies
**Interventions**	Treatment of COVID-19 with oral antivirals No treatment of COVID-19	Treatment with ancillary medications or medications not recommended for COVID-19 treatment such as corticosteroids, ivermectin, and anticoagulation.
**Outcomes**	Prevalence, hospitalizations, deaths, resistance, recovery, and immune response	Adverse events and side effects

* The CDC library conducted a search for studies published during February 1, 2020–November 29, 2023 using the following terms: “Paxlovid rebound,” “SARS-CoV-2 viral rebound,” “SARS-CoV-2 rebound,” “nirmatrelvir/ritonavir rebound,” “molnupiravir rebound,” “SARS-CoV-2 infection rebound,” “SARS-CoV-2 viral load rebound,” “rebound phenomenon,” “SARS-CoV-2 viral kinetics,” “SARS-CoV-2 virologic rebound,” and “SARS-CoV-2 clinical rebound.” Databases queried were PubMed, JSTOR, and Google Scholar.

**TABLE 2:** Summary of seven SARS-CoV-2 rebound studies among persons who did and did not receive antiviral treatment with nirmatrelvir/ritonavir or molnupiravir — February 1, 2020–November 29, 2023*

Study author, year	Study type	Definition of rebound	Sample size	Treatment	Rebound prevalence, % (no./No.)		p-value	Study conclusions and key limitations
					With treatment	Without treatment		
Anderson et al., 2022^†^	RCT	0.5-log increase in viral load on day 10 or day 14 if only one value was available or on days 10 and 14 if both values were available	2,216	N/R	2.3 (23/990)	1.7 (17/980)	0.34	Similar incidence of viral load rebound in N/R and placebo groups
								Viral load rebound not retrospectively associated with low nirmatrelvir exposure; recurrence of moderate to severe symptoms, or development of nirmatrelvir resistance
								Limitations: only unvaccinated persons included in study, conducted during pre-Omicron period, viral load determined by PCR, does not translate directly to the presence of infectious virus, and is not perfectly correlated with current or new clinical symptoms
Edelstein et al., 2023^§^	PC	Def 1: a positive SARS-CoV-2 viral culture result after a previous negative test result	127	N/R	21 (15/72)	2 (1/55)	0.001	Viral rebound associated with N/R use
		Def 2: combination of a nadir viral load below 4.0 log_10_ copies/mL followed by an increase in viral load that was ≥1.0 log_10_ copies/mL above the nadir, and two consecutive viral load results of 4.0 log_10_ copies/mL or higher						Limitations: not RCT; significant differences between those taking N/R and untreated persons (e.g., number of COVID-19 vaccinations, older, and immunosuppression)
Pandit et al., 2023^¶^	PC	Positive rapid antigen test result after a negative antigen test result and symptom rebound	N/R = 127; control = 43	N/R	14 (18/127)	9 (4/43)	0.41	Rebound after clearance of test result positivity or symptom resolution is higher than previously reported
								Limitations: not RCT
Smith-Jeffcoat et al., 2023**	Prospective/propensity score matching	Symptom rebound was defined as an increase of at least two symptoms any time after treatment completion or proxy. Viral load rebound was defined as an increase of ≥1 log_10_ IU/mL (increasing to or above 5.0 log_10_ IU/mL) any time after treatment	1,234	N/R	Symptom rebound: 32 (41/130)	Symptom rebound: 20 (47/241)	0.009	Patients completing N/R treatment experienced fewer symptoms and lower viral load but rebound occurred more often compared with untreated persons; providers should prescribe N/R, when indicated, and communicate rebound risk to patients
					Viral load rebound: 27 (26/130)	Viral load rebound: 7 (12/241)	<0.001	Limitations: not RCT; daily symptoms and viral load were only available for 10 days after enrollment; unmeasured differences between N/R-treated and untreated participants
Tadmor et al., 2023^††^	RC (EMR)	Positive PCR test result after negative test result	331	N/R	9.0 (8/89)	3.6 (8/219)	0.05	Higher incidence of rebound in patients with CLL treated for SARS-CoV-2 with N/R or molnupiravir compared with nontreated CLL patients or nonleukemia high-risk patients
				molnupiravir	8.7 (2/23)		0.24	Limitations: not RCT
Wong et al., 2023^§§^	RC	Reduction in Ct value (≥3) on quantitative RT-PCR test between 2 consecutive measurements, with decrease sustained in an immediately subsequent Ct measurement (for those patients with ≥3 Ct measurements)	4,592	N/R	6.6; 95% CI = 4.1–10.5 (6/242)	4.5; 95% CI = 3.9–5.2 (170/3,787)	0.13	Viral rebound rates were similar between patients with and without antiviral treatment
				molnupiravir	4.8; 95% CI = 3.3–6.9 (27/563)		0.75	Viral burden rebound not associated with adverse clinical outcomes
								Limitations: not RCT
Wong et al., 2022^¶¶^	RC	Def 1: Ct >40, decreased to ≤40	12,629	N/R	Def 1: N/R: 1.0	Def 1: 0.6	Def 1: 0.56	Low incidences of viral rebound in molnupiravir users, N/R users, and antiviral nonusers among patients with COVID-19
		Def 2: Ct >36, decreased to ≤36			Def 2: N/R: 4.6	Def 2: 4.4	Def 2: 0.95	Viral rebound is not associated with higher mortality in antiviral users
		Def 1: Ct>40, decreased to ≤40		molnupiravir	Def 1: molnupiravir: 0.8	Def 1: 0.6	Def 1: 0.56	
		Def 2: Ct >36, decreased to ≤36			Def 2: molnupiravir: 4.6	Def 2: 4.4	Def 2: 0.95	Limitations: not RCT

**Abbreviations:** CLL = chronic lymphocytic leukemia; Ct = cycle threshold; Def = definition; EMR = electronic medical records; N/R = nirmatrelvir/ritonavir; PC = prospective cohort; RC = retrospective cohort; RCT = randomized controlled trial; RT-PCR = real-time polymerase chain reaction.

* From PubMed, JSTOR, and Google Scholar databases, 303 publications published during February 1, 2020–November 29, 2023, were identified; nine duplicate citations were removed. Among the 294 abstracts reviewed, 229 irrelevant abstracts were removed (i.e., not COVID-19–related or examined another aspect of COVID-19). Among the 65 relevant publications determined to be eligible for detailed review, 42 publications were removed because they did not meet inclusion criteria (i.e., preprints, editorials, case reports, or studies of ancillary medications). Overall, 23 publications were included in the review including these seven publications that compared rates of rebound among persons with and without use of COVID-19 antivirals.

^†^ https://doi.org/10.1056/NEJMc2205944

^§^ https://doi.org/10.7326/M23-1756

^¶^ https://doi.org/10.1093/cid/ciad102

** https://doi.org/10.1093/cid/ciad696

^††^ https://doi.org/10.1080/10428194.2023.2183732

^§§^ https://doi.org/10.1016/S1473-3099(22)00873-8

^¶¶^ https://doi.org/10.1001/jamanetworkopen.2022.45086

## Review Findings

### Studies of Rebound in Patients Who Did and Did Not Receive Antiviral Treatment

SARS-CoV-2 rebound with and without the use of antiviral treatment were described in previous studies (*10*–*16*). The definition of and methods assessing SARS-CoV-2 rebound, including frequency and duration of specimen collection, varied among studies (Table 2). No hospitalizations or deaths were reported among outpatients who experienced rebound, because symptoms were mild.

Four retrospective cohort studies found similar frequencies of viral rebound among persons who did and did not receive COVID-19 antiviral treatment (*10*,*12*,*15**–**16*). Three studies found higher frequencies of rebound among treated persons: the first study examined persons with chronic lymphocytic leukemia (*14*); the second examined treated persons who were older (median age = 57 years versus 39 years; p<0.001), received more COVID-19 vaccine doses (4 versus 3; p<0.001), and had higher rates of immunosuppression (32% versus 9%; p<0.001) than did untreated persons (*11*); and the third used propensity score matching to ensure the treated and untreated groups were well matched, but had limited follow-up time (*13*).

A large retrospective, observational study found similar rates of rebound and no statistically significant differences among patients treated with nirmatrelvir/ritonavir (6.6%; 95% CI = 4.1%–10.5%), molnupiravir (4.8%; 95% CI = 3.3%–6.9%) and those who received no treatment (4.5%; 95% CI = 3.9%–5.2%) (Table 2) (*15*). Persons with immunocompromising conditions had higher odds of viral rebound regardless of treatment status: nirmatrelvir/ritonavir (odds ratio [OR] = 7.37; 95% CI = 2.56–21.26), molnupiravir (OR = 3.05; 95% CI = 1.28–7.25), and no treatment (OR = 2.21; 95% CI = 1.50–3.27). Among patients receiving nirmatrelvir/ritonavir, the odds of virologic rebound were higher among those aged 18–65 years compared with those aged >65 years (OR = 3.09; 95% CI = 1.00–9.53), those with high comorbidity prevalence (score >6 on the Charlson Comorbidity Index [OR = 6.02; 95% CI = 2.09–17.38]), and those concomitantly taking corticosteroids (OR = 7.51; 95% CI = 1.67–33.82), whereas the odds were lower among those who were not fully vaccinated (OR = 0.16; 95% CI = 0.04–0.67).

Initial analysis of EPIC-HR trial data showed that viral rebound rates were low and similar between the treated and untreated groups (Table 2) (*10*). In addition, rebound was not associated with low nirmatrelvir/ritonavir levels, hospitalization or death, severe symptom relapse, vaccination or serologic status, or emergent mutations (*8*,*10*).

### Infectivity, Resistance, and Immune Response

One observational study demonstrated that duration of shedding of infectious virus was longer among persons with rebound (14 days) compared with those without rebound (3 days), but found no evidence of resistance-associated mutations using genomic sequencing (*11*). Another study of biomarkers among six patients with rebound after treatment with nirmatrelvir/ritonavir demonstrated that a robust immune response was present during rebound, likely reducing risk for disease progression (*17*). This study also found no evidence of resistance.

### Onset and Duration of Rebound

**FIGURE 2:**
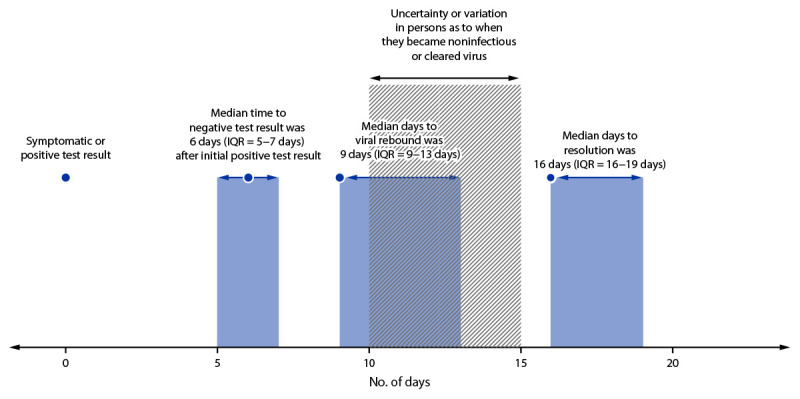
Timing of viral rebound and resolution during SARS-CoV-2 infection among 22 patients*^,†^ — February 1, 2020–November 29, 2023 * Median time to negative test result was defined as day of first negative viral test result (polymerase chain reaction or antigen) after initial positive test result. Viral rebound was defined as the first positive viral test result (polymerase chain reaction or antigen) after a negative test result. Resolution was defined as first negative viral test result after day 1 of viral rebound. ^†^ Timing and duration of viral rebound generated using data from 22 patients in three studies that used a virologic definition of rebound and had complete data: https://doi.org/10.1093/cid/ciac512, https://doi.org/10.1056/NEJMc2206449, and https://doi.org/10.1016/j.jinf.2022.06.011.

Among 22 patients (from three studies) with available virologic data and who received treatment, median time to negative test results was 6 days (IQR = 5–7 days) after initial positive test result (*18*–*20*) (Figure 2). Median time to viral rebound was 9 days (IQR = 9–13 days) after diagnosis, and to resolution was 16 days (IQR = 16–19 days) into the viral illness. Rebound occurred during the course of illness when there was variability in viral load because of host factors (*21*).

## Discussion

Current evidence, including randomized controlled trial and observational data, suggests that SARS-CoV-2 rebound occurs initially as a mild illness 3–7 days after resolution of the initial acute illness, occurs in both treated and untreated patients, and is not associated specifically with receiving nirmatrelvir/ritonavir. Moreover, rebound occurs when there is variable, host-mounted immune response to infection during the course of illness. Finally, no hospitalizations or deaths were reported among outpatients who experienced rebound.

Some observational studies demonstrated a higher frequency of rebound among treated persons (10%–14%) (*11*,*14*,*22*) than reported by the randomized controlled trial, EPIC-HR (*8*,*10*) (Supplementary Table, https://stacks.cdc.gov/view/cdc/137156). Viral rebound might occur in persons on antiviral treatment because they are at high risk for severe disease and might have host factors, such as immunosuppression, that contribute to the natural variability in viral dynamics (*21*). Risk factors for rebound appear to be similar to risk for severe disease, but further studies are needed to understand whether persons with certain characteristics or underlying medical conditions are predisposed to experiencing rebound. Another important consideration is that persons receiving antiviral treatment might be at higher risk for experiencing rebound given the viral suppression related to use of treatment early in the disease course and resumption of viral replication after completion of treatment because of delayed viral clearance. This elevated risk could be due to early discontinuation of antiviral treatment or the need for longer courses of treatment among certain persons, such as those who are immunocompromised (*14*). Two ongoing clinical trials of nirmatrelvir/ritonavir will further characterize the frequency of rebound after different durations of nirmatrelvir/ritonavir treatment among immunocompromised subjects^¶¶^ and the potential benefit of nirmatrelvir/ritonavir retreatment among subjects with posttreatment rebound.***

Rebound does not likely represent reinfection or resistance to treatment (*12*); however, further studies are needed to confirm this finding. The FDA analysis identified potential treatment-associated mutations that were not clinically relevant among two treated patients because rebound symptoms resolved without hospitalization (*8*). It is important to ensure that use of antivirals does not accelerate viral evolution and result in resistant mutations, such as through counseling patients to complete antiviral treatment and monitoring for resistance using molecular analyses. Two studies demonstrated shedding of infectious virus during rebound (*8*,*11*). Comparisons of genomic strains present in both acute and rebound episodes and viral culture to determine infectiousness are important to understanding the clinical implications of rebound. In addition, a large assessment of innate and adaptive immunity and monitoring biomarkers of inflammation and cytokine storm would contribute to understanding of the underlying pathophysiology of recurrence.

### Limitations

The findings in this report are subject to at least five limitations. First, standardized definitions for symptom, viral, and clinical rebound were not used across studies. Using standard definitions to accurately reflect outcomes could improve interpretability and comparisons of data across studies and settings. Most studies examined symptom or viral rebound. A definition that requires reemergence of virus after complete resolution of illness, which takes 7–10 days for a healthy adult, and a negative viral test result after resolution of initial symptoms would allow for examination of clinical implications of rebound or recrudescence, such as a dysregulated immune response (*23*). Second, publications about recurrences and viral kinetics might have been missed given the narrow search. Third, a major limitation of observational studies is the difficulty in verifying whether antiviral treatment courses were completed and whether vaccination status and previous infection were documented accurately. Fourth, few studies correlated symptoms with viral load, which makes the significance of recurrence of mild symptoms difficult to understand because symptoms are subjective and might not represent viral reactivation. Finally, ascertainment bias is also possible given that persons receiving antiviral treatment are closely followed, and more likely to report recurrent symptoms, which would explain the early case reports being associated with nirmatrelvir/ritonavir, the most commonly used oral antiviral in the United States.

### Implications for Public Health Practice

Viral rebound can occur in persons who do and do not receive antiviral treatment and might reflect viral fluctuation that is part of the natural disease process early in the course of illness. Risk for experiencing rebound could be related to many factors, such as immunosuppression, delayed viral clearance, and overall immune response. The current literature review, along with a recently published randomized trial (*8*), suggests the substantial benefit of antiviral treatment among persons at risk for severe disease outweighs the risk for rebound, because rebound resolves quickly and is not associated with an increase in severity of recurring signs and symptoms. Increased education and awareness among practitioners and patients about rebound not increasing risk for hospitalization or death might increase use of COVID-19 treatment. According to NIH COVID-19 Treatment Guidelines, rebound should not deter providers from prescribing life-saving antiviral treatments when indicated to prevent morbidity and mortality from COVID-19 (*1*).

## Footnotes

* https://covid.cdc.gov/covid-data-tracker/#datatracker-home (Accessed December 15, 2023).

†
https://www.cdc.gov/vaccines/covid-19/clinical-considerations/covid-19-vaccines-us.html

§
https://www.medscape.com/viewarticle/987121?form=fpf

¶
https://stacks.cdc.gov/view/cdc/117609

**
https://www.fda.gov/news-events/press-announcements/fda-approves-first-oral-antiviral-treatment-covid-19-adults

†† Relevant publications were identified based on the titles and abstracts using EndNote (version 20; Clarivate). Two reviewers independently screened all titles and abstracts and hand-searched references of retrieved publications. Disagreements were discussed, and duplicates were removed. Variables extracted from publications included date published, country of study, journal, study design, patient median age, sex, COVID-19 vaccination status, definition of rebound, duration of rebound symptoms, sample size, antiviral treatment type, rebound prevalence, outcome, study limitations, and conclusions.

§§ 45 C.F.R. part 46.102(l)(2), 21 C.F.R. part 56; 42 U.S.C. Sect. 241(d); 5 U.S.C. Sect. 552a; and 44 U.S.C. Sect. 3501 et seq.

¶¶
https://clinicaltrials.gov/ct2/show/NCT05438602

***
https://clinicaltrials.gov/study/NCT05567952?cond=post-treatment%20rebound%20COVID-19&rank=1
